# Role of dietitians in optimizing medical nutrition therapy in cardiac surgery patients: A secondary analysis of an international multicenter observational study

**DOI:** 10.1002/jpen.2755

**Published:** 2025-04-06

**Authors:** Ellen Dresen, Danielle E. Bear, Ashley DePriest, Ranna Modir, Omy Naidoo, Charlene Compher, Andrea Ho, Pui Hing Foong, Maria Eloisa Garcia Velásquez, Zheng‐Yii Lee, Charles Chin Han Lew, Gunnar Elke, Jayshil J. Patel, Liam McKeever, Katharina Berschauer, Catarina Rosa Domingues, Juan Carlos Lopez‐Delgado, Patrick Meybohm, Daren K. Heyland, Christian Stoppe

**Affiliations:** ^1^ University Hospital Würzburg Department of Anaesthesiology, Intensive Care, Emergency and Pain Medicine Würzburg Germany; ^2^ Guy's and St. Thomas' NHS Foundation Trust Department of Nutrition and Dietetics London UK; ^3^ Guy's and St. Thomas' NHS Foundation Trust Department of Critical Care London UK; ^4^ Emory University Hospital Department of Food and Nutrition Services Atlanta Georgia USA; ^5^ Stanford University Medical Center Department of Clinical Nutrition Stanford California USA; ^6^ PMB Healthcare Centre Newtricion Wellness Dieticians Pietermaritzburg South Africa; ^7^ University of Pennsylvania School of Nursing and Hospital of the University of Pennsylvania Philadelphia Pennsylvania USA; ^8^ University of Toronto, Sunnybrook Health Sciences Centre Departments of Cardiac Surgery and Critical Care Medicine Toronto Ontario Canada; ^9^ National Heart Institute Dietetics & Food Services Kuala Lumpur Malaysia; ^10^ San Francisco Clinic Hospital Department of Clinical Nutrition Guayaquil Ecuador; ^11^ University of Malaya, Faculty of Medicine Department of Anaesthesiology Kuala Lumpur Malaysia; ^12^ Deutsches Herzzentrum der Charité Department of Cardiac Anesthesiology and Intensive Care Medicine Berlin Germany; ^13^ Charité — Universitätsmedizin Berlin, Corporate Member of Freie Universität Berlin and Humboldt‐Universität zu Berlin Berlin Germany; ^14^ Ng Teng Fong General Hospital Department of Dietetics & Nutrition Singapore Singapore; ^15^ Singapore Institute of Technology Faculty of Health and Social Sciences Singapore Singapore; ^16^ University Medical Center Schleswig‐Holstein Department of Anesthesiology and Intensive Care Medicine Kiel Germany; ^17^ Medical College of Wisconsin Division of Pulmonary and Critical Care Medicine, Department of Medicine Milwaukee Wisconsin USA; ^18^ Rush University Medical Center Department of Clinical Nutrition Chicago Illinois USA; ^19^ Centro Hospitalar Universitário de Lisboa Central EPE Medical Emergency Unit Lisbon Portugal; ^20^ Hospital Clinic of Barcelona Barcelona Spain; ^21^ University of Barcelona, School of Nursing Departament d'Infermeria Fonamental i Médico‐Quirúrgica Barcelona Spain; ^22^ Queen's University Department of Critical Care Medicine Kingston Ontario Canada

**Keywords:** adherence, cardiac surgery, clinical practice, dietetic services, dietitians, energy, medical nutrition therapy, protein

## Abstract

**Background:**

Better understanding the impact of dietetic services on nutrition practices seems required as it may represent an opportunity for optimization in post–cardiac surgery patients. The present study aims to evaluate and compare nutrition practices and clinical outcomes in post–cardiac surgery intensive care unit (ICU) patients with and without dietetic services.

**Methods:**

This is a secondary analysis of a multinational prospective observational study in patients (*n* = 237) with >72 h of post–cardiac surgical ICU stay with and without dietetic services describing nutrition practices and outcomes up to 12 days after ICU admission.

**Results:**

Dietetic services were available in 61.5% (8 of 13) ICUs (1.0 ± 0.5 full‐time equivalents/10 beds). Enteral nutrition was initiated <48 h from ICU admission in 49.6% and 59.1% of patients at sites with vs without dietetic services, respectively. Parenteral nutrition was started within 118.3 ± 56.5 and 131.5 ± 69.2 h at sites with vs without dietetic services, respectively. Energy target (23.7 ± 4.8 vs 24.6 ± 4.8 kcal/kg body weight/day) and actual supply (10.5 ± 6.7 vs 10.3 ± 6.2 kcal/kg body weight/day) did not differ between the groups. Protein targets (1.4 ± 0.4 vs 1.1 ± 1.3 g/kg body weight/day) and actual protein provision (0.6 ± 0.4 vs 0.4 ± 0.3 g/kg body weight/day) were higher in patients at sites with vs without dietetic services.

**Conclusion:**

Improvements in medical nutrition therapy practices in patients after cardiac surgery are needed in ICUs with and without dietetic services. Appropriately staffed dietetic services as essential members of the medical care team may be crucial to transfer knowledge on adequate medical nutrition therapy strategies into practice.

**Abbreviations**
APACHE: Acute Physiology and Chronic Health Evaluation
ICU: intensive care unit
IQR: interquartile range
mNUTRIC: modified Nurition Risk in Critically Ill
SD: standard deviation
SOFA: Sequential Organ Failure Assessment

## CLINICAL RELEVANCY STATEMENT

Patients after cardiac surgery are known to receive delayed nutrition support, and defined energy and protein targets are not adequately covered by actual provision. However, evidence on appropriate medical nutrition therapy strategies (eg, the optimal timing and dosage of energy and protein supply) in concerning patients is limited and further research urgently warranted. Besides, further efforts to strengthen the role and staffing of dietitians in the treatment of high‐risk patients, such as those after cardiac surgery, are required to transfer knowledge on appropriate medical nutrition therapy strategies gained by future research into practice.

## INTRODUCTION

Individualized, disease‐specific and phase‐specific medical nutrition therapy is a key component of the management of critically ill patients, particularly in those at heightened risk for complex and prolonged stays in intensive care units (ICU).^1^ Pre‐existing malnutrition is frequent in post–cardiac surgery patients, and they are susceptible to prolonged ICU stay, which itself begs a risk of iatrogenic malnutrition.^2^ Unfortunately, post–cardiac surgery patients, compared with noncardiac surgical and medical ICU patients, receive less nutrition provision during their ICU stay.^3^ Observational data revealed that nutrition support in patients after cardiac surgery was initiated late, and defined energy and nutrient targets were not met by actual provision.^4^ Furthermore, post–cardiac surgery patients have marked perioperative inflammatory responses and undergo profound metabolic, endocrinologic, and immunologic alterations throughout their ICU stay;^5^ and inadequate receipt of nutrition may be an additive risk factor for acquired postoperative malnutrition.

Dietitians are uniquely positioned to provide insights into various aspects of medical nutrition therapy contributing to interdisciplinary treatment approaches aimed at optimizing nutrition care.^6^, ^7^ However, dietetic services are not universally integrated in the critical care setting. This is evidenced by previous studies examining medical nutrition therapy practices in ICUs worldwide.^8^, ^9^ Based on existing evidence, a better understanding of the impact of dietetic services on nutrition practices seems urgently required as it may represent an opportunity to optimize medical nutrition therapy in post–cardiac surgery patients.

To address this gap, a secondary analysis of a prospective, multinational observational study was conducted. The aim of the present study was to evaluate and compare nutrition practices and clinical outcomes in post–cardiac surgery ICU patients with and without dietetic services.

## PATIENTS AND METHODS

### Ethics statement

The research ethics committees at Aachen University and Queens University approved the International Nutrition Survey 2018 (Ethics Committee file no.: EK297/17, Ethics Committee Aachen, approval given on November 17, 2017; ROMEO/TRAQ: #6004791, Department Code: DMED‐984‐06 Ethic Board Queens University, approval given on July 28, 2017).

### Study design and participants

This is a secondary analysis of a multinational prospective observational study in patients (*n* = 237) with >72 h of post–cardiac surgical ICU stay with and without dietetic services describing nutrition practices and outcomes up to 12 days after ICU admission. The detailed methodology of the International Nutrition Survey 2018 has been published elsewhere.^4^ In brief, patients were recruited from ICUs of medical centers having a minimum of eight beds and dietetic services or an intensivist familiar with clinical nutrition to complete data collection. A total of 20 patients per site were enrolled consecutively with the following inclusion criteria: (1) age ≥18 years, (2) admitted to the ICU immediately following a cardiac surgical procedure, (3) expected ICU length of stay ≥72 h, and (4) mechanical ventilation upon ICU admission or within 48 h after ICU admission. Given the observational nature of the International Nutrition Survey 2018, the estimation/determination of nutrition requirements (eg, energy and protein targets), feeding practices, and metabolic monitoring (eg, blood glucose) were not protocolized and instead were carried out based on the local standards of the participating sites.

### Data collection

Severity of illness at baseline was assessed using Acute Physiology and Chronic Health Evaluation II (APACHE II) and Sequential Organ Failure Assessment (SOFA). Additionally, baseline nutrition risk was determined using the modified Nutrition Risk in the Critically Ill (mNUTRIC) tool.^10^, ^11^

Data related to medical nutrition therapy were collected daily from ICU admission up to a maximum of 12 days in the ICU including the route of nutrition support (oral nutrition, enteral nutrition, parenteral nutrition, or combined enteral + parenteral/supplemental parenteral nutrition), prescribed energy and protein (target determined at baseline), and actual provision.

Other nutrition‐related information was also collected, that is, blood glucose concentration, insulin dose, use of prokinetics, head of the bed elevation, composition of enteral nutrition products, type of parenteral lipids used, and reasons for enteral nutrition feeding interruptions.

Clinical outcomes (duration of mechanical ventilation, ICU and hospital length of stay, and mortality) were censored at day 60. Data extracted from patients’ records were entered online using a secure web‐based data collection tool.^4^

### Statistical analyses

Site characteristics as well as patient demographic and clinical data are described using mean and standard deviation (SD), median and interquartile range (IQR), and counts (percentages). Data of medical nutrition therapy and metabolic monitoring are reported as mean (SD), median (IQR), and count (percentage).

Based on the collected raw data on nutrition practices, the percentage of defined energy and protein targets being met by actual provision (from either enteral nutrition and/or parenteral nutrition and propofol) throughout the study period was calculated. Days without any nutrition (neither enteral nutrition nor parenteral nutrition) during the 12 days were included, counting as 0.0% of targets met, and days after progression to exclusive oral intake were excluded.^12^ Further, the number of patients who met >80.0% of the defined energy and protein targets by actual provision on day 7 was analyzed.

In general, all the data are analyzed and compared between sites with vs without dietetic services. All analyses were performed using SAS 9.4 (SAS Institute Inc, Cary, NC, USA).

## RESULTS

### Site and patient characteristics

A total of 13 ICUs from six countries across three continents participated in the International Nutrition Survey 2018, enrolling a total of 237 patients. Of the participating sites, eight (61.5%) had dietetic services at the ICU, with a mean ± SD of 1.0 ± 0.5 full‐time positions for dietetic services needed per 10 beds. Sites with dietetic services were smaller (24.3 ± 6.5 ICU beds) compared with sites without dietetic services (50.4 ± 27.7 ICU beds). Nutrition protocols were used in four (50.0%) and three (60.0%) of the sites with and without dietetic services, respectively. Table S1 describes the characteristics of the participating sites.

A total of 140 and 97 patients were treated in ICUs with and without dietetic services, respectively. Patients at sites with and without dietetic services had high severity of illness indicated by APACHE II (22.9 ± 7.0 and 20.0 ± 7.8) and SOFA score (10.9 ± 3.3 and 9.0 ± 4.0) and were at high nutrition risk (mNUTRIC 6.0 ± 1.5 and 5.4 ± 1.8) (Table 1). History of reduced oral intake or weight loss was more complete at sites with dietetic services than at sites without (“Do not know”: 20.0% vs 35.1%) (Table 1). Cardiac surgery–specific patient baseline characteristics are shown in Table S2.

**Table 1:** Baseline characteristics.

	Patients at sites with dietetic services	Patients at sites without dietetic services
	(*n* = 140)	(*n* = 97)
Age, years		
Mean ± SD	62.3 ± 14.2	64.3 ± 13.7
Height, m		
Mean ± SD	1.7 ± 0.1	1.7 ± 0.1
Dry body weight, kg		
Mean ± SD	78.3 ± 21.2	81.0 ± 20.8
BMI, kg/m^2^		
Mean ± SD	27.5 ± 6.8	27.7 ± 6.0
mNUTRIC score		
Mean ± SD	6.0 ± 1.5	5.4 ± 1.8
APACHE II score		
Mean ± SD	22.9 ± 7.0	20.0 ± 7.8
SOFA		
Mean ± SD	10.9 ± 3.3	9.0 ± 4.0
Patients who had a history of reduced oral intake or weight loss, *n* (%)		
Yes	13 (9.3)	1 (1.0)
No	99 (70.7)	62 (63.9)
Do not know	28 (20.0)	34 (35.1)

Abbreviations: APACHE II, Acute Physiology and Chronic Health Evaluation II; BMI, body mass index; mNUTRIC, modified Nutrition Risk in Critically Ill; SD, standard deviation; SOFA, Sequential Organ Failure Assessment.

### Nutrition therapy

In patients at sites with and without dietetic services, exclusive enteral nutrition was the predominant route of medical nutrition therapy (*n* = 112 [80.0%] vs *n* = 75 [77.3%]). Exclusive parenteral nutrition was used in one (0.7%) patient at a site with dietetic services, whereas none of the patients at sites without dietetic services received this nutrition strategy. Combined enteral plus parenteral nutrition was used in 15 (10.7%) vs 18 (18.6%) patients at sites with and without dietetic services, respectively (Table 2).

**Table 2:** Feeding performance measures.

	Patients at sites with dietetic services	Patients at sites without dietetic services
	(*n* = 140)	(*n* = 97)
Type of nutrition, *n* (%)		
Enteral nutrition only	112 (80.0)	75 (77.3)
Parenteral nutrition only	1 (0.7)	0 (0.0)
Enteral plus parenteral nutrition	15 (10.7)	18 (18.6)
None	12 (8.6)	4 (4.1)
Initiation of enteral nutrition		
Mean ± SD hours from admission to start of enteral nutrition	57.3 ± 42.8	48.1 ± 38.7
Median (IQR) hours from admission to start of enteral nutrition	48.0 (26.2–74.1)	44.2 (18.8–68.0)
Initiation of enteral nutrition <48 h after ICU admission		
*n/N* (%) of patients	63/127 (49.6)	55/93 (59.1)
Initial enteral nutrition delivery order, *n* (%) of patients		
Different types		
Initiate enteral nutrition: start at low rate and progress to hourly goal rate	81 (57.9)	33 (34.0)
Initiate enteral nutrition: keep a low rate (trophic feeds: no progression)	13 (9.3)	11 (11.3)
Initiate enteral nutrition: bolus feeds	16 (11.4)	8 (8.2)
Initiate enteral nutrition: start at hourly goal rate	8 (5.7)	0 (0.0)
Keep at nothing by mouth	1 (0.7)	20 (20.6)
Initiate enteral nutrition: start at or progress to 24 h volume goal‐based hourly rate	5 (3.6)	10 (10.3)
Oral nutrition	13 (9.3)	14 (14.4)
Parenteral nutrition	3 (2.1)	1 (1.0)
Location of feeding tube, *n* (%) of patient days in stomach		
Different types		
Gastric	958 (64.9)	855 (82.1)
Small bowel	156 (10.6)	5 (0.5)
No tube in place	293 (19.9)	132 (12.7)
Use of motility agents, *n* (%) of patient days		
Different types		
Metoclopramide	99 (6.7)	143 (13.7)
Erythromycin	13 (0.9)	34 (3.3)
Methylnaltrexon	2 (0.1)	0 (0.0)
Domperidone	9 (0.6)	0 (0.0)
Other	5 (0.3)	0 (0.0)
Initiation of parenteral nutrition		
Mean ± SD hours from admission to start of parenteral nutrition	118.3 ± 56.5	131.5 ± 69.2
Median (IQR) hours from admission to start of parenteral nutrition	106.9 (79.4–146.8)	126.4 (63.0–190.5)

Abbreviations: ICU, intensive care unit; IQR, interquartile range; SD, standard deviation.

The mean ± SD time to enteral nutrition initiation was 57.3 ± 42.8 h in patients at sites with dietetic services compared with 48.1 ± 38.7 h in patients at sites without dietetic services (Table 2). In patients admitted to sites with and without dietetic services, respectively, the initiation of enteral nutrition <48 h from ICU admission was achieved in 49.6% and 59.1% of patients (Table 2).

With regard to the initial enteral nutrition delivery order, differences have been observed between patients at sites with and without dietetic services. As an example, starting at a low rate and progressively increasing to the hourly goal rate was more often used in patients at sites with dietetic services (*n* = 81; 57.9%) compared with patients at sites without dietetic services (*n* = 33; 34.0%; Table 2). The location of feeding tubes in the small bowel was more often used in patients at sites with compared with without dietetic services (*n* = 156 vs *n* = 5 of patient days; 10.6% vs 0.5% of patient days) (Table 2). With one exception, the use of different motility agents was more frequent in patients admitted to sites without dietetic services compared with patients at sites with dietetic services (Table 2).

### Energy and protein delivery performance metrics

In patients at sites with vs without dietetic services, defined total energy targets (23.7 ± 4.8 vs 24.6 ± 4.8 kcal/kg body weight/day) and actual energy delivery via enteral plus parenteral nutrition plus propofol (10.5 ± 6.7 vs 10.3 ± 6.2 kcal/kg body weight/day) were similar (Table 3).

**Table 3:** Overall performance (patient level).

	Patients at sites with dietetic services	Patients at sites without dietetic services
Overall performance	(*n* = 140)	(*n* = 97)
Total energy target, kcal/day		
Mean ± SD	1,796.2 ± 357.2	1,925.4 ± 311.1
Median (IQR)	1,757.0 (1,532.5–1,972.0)	1,900.0 (1,700.0–2,075.0)
Total energy target, kcal/kg body weight/day		
Mean ± SD	23.7 ± 4.8	24.6 ± 4.8
Median (IQR)	25.0 (20.6–26.4)	25.0 (21.5–25.0)
Total protein target, g/day		
Mean ± SD	104.9 ± 31.5	85.0 ± 16.8
Median (IQR)	100.0 (82.0–120.0)	83.0 (73.2–94.5)
Total protein target, g/kg body weight/day		
Mean ± SD	1.4 ± 0.4	1.1 ± 1.3
Median (IQR)	1.3 (1.2–1.6)	1.0 (1.0–1.2)
Total received energy (enteral nutrition + parenteral nutrition + propofol), kcal/day		
Mean ± SD	778.3 ± 504.6	786.9 ± 410.9
Median (IQR)^a^	712.3 (362.3–1,055.7)	729.2 (499.6–1,015.4)
Total received energy (enteral nutrition + parenteral nutrition + propofol), kcal/kg body weight/day		
Mean ± SD	10.5 ± 6.7	10.3 ± 6.2
Median (IQR)^a^	9.5 (5.3–14.9)	9.3 (6.1–13.0)
Total received protein (enteral nutrition + parenteral nutrition), g/day		
Mean ± SD	43.3 ± 30.8	28.7 ± 18.6
Median (IQR)^a^	38.3 (20.6–63.4)	24.6 (15.5–39.4)
Total received protein (enteral nutrition + parenteral nutrition), g/kg body weight/day		
Mean ± SD	0.6 ± 0.4	0.4 ± 0.3
Median (IQR)^a^	0.5 (0.3–0.8)	0.3 (0.2–0.5)
Received energy from enteral nutrition (enteral nutrition‐only patients), kcal/day		
Mean ± SD	704.4 ± 447.1	624.7 ± 339.8
Median (IQR)^b^	669.0 (342.9–1,007.4)	598.3 (375.0–800.0)
Received energy from enteral nutrition (enteral nutrition‐only patients), kcal/kg body weight/day		
Mean ± SD	9.4 ± 5.9	8.3 ± 5.2
Median (IQR)^b^	8.9 (4.7–13.3)	7.6 (5.0–10.3)
Received protein from enteral nutrition (enteral nutrition‐only patients), g/day		
Mean ± SD	43.1 ± 29.7	25.6 ± 15.4
Median (IQR)^b^	35.8 (20.4–59.3)	22.9 (14.2–33.7)
Received protein from enteral nutrition (enteral nutrition‐only patients), g/kg body weight/day		
Mean ± SD	0.6 ± 0.4	0.3 ± 0.2
Median (IQR)^b^	0.5 (0.3–0.8)	0.3 (0.2–0.4)
Total adequacy of energy (enteral nutrition + parenteral nutrition + propofol), %		
Mean ± SD	44.6 ± 26.9	42.3 ± 24.1
Median (IQR)^a^	42.9 (23.3–63.4)	38.7 (24.8–57.1)
Total adequacy of protein (enteral nutrition + parenteral nutrition), %		
Mean ± SD	41.7 ± 26.8	35.6 ± 26.4
Median (IQR)^a^	39.9 (20.0–65.0)	29.4 (17.0–50.4)
Adequacy of energy from enteral nutrition (enteral nutrition‐only patients), %		
Mean (SD)	40.3 ± 23.6	33.8 ± 20.0
Median (IQR)^b^	38.9 (20.9–55.1)	31.3 (18.9–44.9)
Adequacy of protein from enteral nutrition (enteral nutrition‐only patients), %		
Mean (SD)	41.9 ± 25.8	31.4 ± 20.8
Median (IQR)^b^	39.4 (20.8–65.0)	28.6 (15.9–39.4)
Total adequacy of energy (enteral nutrition + parenteral nutrition + propofol) >80.0% on day 7		
*n/N* (%) of patients	51/110 (46.4)	19/79 (24.1)
Total adequacy of protein (enteral nutrition+parenteral nutrition) >80.0% on day 7		
*n/N* (%) of patients	49/110 (44.5)	15/79 (19.0)

Abbreviations: IQR, interquartile range; SD, standard deviation.

^a^ *n* = 233.

^b^ *n* = 220.

Both protein targets (1.4 ± 0.4 vs 1.1 ± 1.3 g/kg body weight/day) and actual protein delivery via enteral plus parenteral nutrition (0.6 ± 0.4 vs 0.4 ± 0.3 g/kg body weight/day) were higher in patients at sites with vs without dietetic services.

In patients receiving exclusive enteral nutrition, actual energy provision was similar in patients at sites with and without dietetic services (9.4 ± 5.9 vs 8.3 ± 5.2 kcal/kg body weight/day, respectively).

Actual protein provision in patients receiving exclusive enteral nutrition at sites with dietetic services was higher than at sites without dietetic services (0.6 ± 0.4 vs 0.3 ± 0.2 g/kg body weight/day; Table 3).

**Figure 1:**
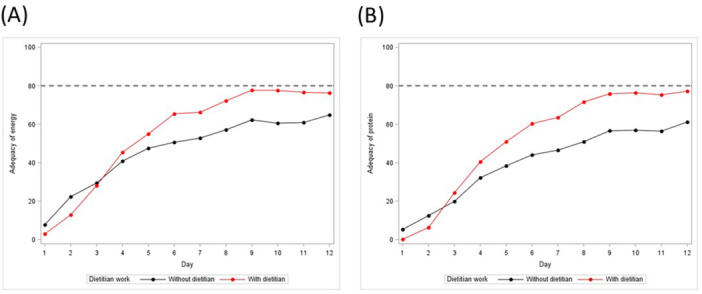
Percentage of (A) energy and (B) protein targets covered by actual provision via enteral nutrition plus parenteral nutrition (plus propofol) in patients at sites with and without dietetic services throughout the study period.

On average, both energy and protein targets had not been reached by actual provision throughout the study period in patients at sites with and without dietetic services, indicated by overall nutrition adequacy (percent of energy and protein targets covered by actual intake) of 44.6% ± 26.9% vs 42.3% ± 24.1% and 41.7% ± 26.8% vs 35.6% ± 26.4%, respectively (Table 3; Figure 1).

However, on day 7, more patients at sites with than without dietetic services reached >80.0% of energy (51/110; 46.4% vs 19/79; 24.1%) and protein targets (49/110; 44.5% vs 15/79; 19.0%), respectively (Table 3).

Further details on absolute values of energy and protein targets and actual supply are shown in Table 3. Site‐level data on nutrition performance in sites with and without dietetic services are shown in Table S3.

### Feeding interruptions

Enteral nutrition feeding interruptions occurred less frequently in patients at sites with compared with without dietetic services for fasting for operating room procedures (1.4% vs 3.0% of patient days), enteral nutrition intolerance (4.4% vs 6.4% of patient days), trial of oral intake (0.4% vs 1.5% of patient days), and inotropes or vasopressor requirement (0.2% vs 2.8% of patient days) (Table S4).

### Use of specific nutrition formulations

Compared with patients at sites without dietetic services, patients at sites with dietetic services more often received fish oil–enriched formulas (0.0% vs 15.0% of patients), whereas polymeric formulas more frequently have been used in patients at sites without vs with dietetic services (100.0% vs 82.7% of patients) (Table S5).

In addition, patients at sites with vs without dietetic services more frequently received soybean oil–based lipids (13.1% vs 2.3% of patients), mixed lipid emulsions composed of medium‐chain triglycerides, soybean oil, olive oil, and fish oil (46.4% vs 7.0%), and lipid‐free nutrition (40.5% vs 0.0% of patients). Patients at sites without vs with dietetic services more often received medium‐ and long‐chain triglyceride mixtures (84.9% vs 0.0%) (Table S6).

### Glycemic control

Glycemic control protocols were implemented at six (75.0%) and three (60.0%) sites with and without dietetic services, respectively (Table S7). Periods of hyperglycemia and hypoglycemia were similar in patients at sites with and without dietetic services (16.0% vs 12.0%; 1.3% vs 0.7%) (Table S7).

### Clinical outcomes

Patients at sites with and without dietetic services had similar mean length of mechanical ventilation (17.8 ± 11.9 vs 16.4 ± 11.3 days), ICU length of stay (20.6 ± 16.1 vs 21.0 ± 17.6 days), and hospital length of stay (28.8 ± 17.1 vs 28.4 ± 17.8 days) (Table 4). No differences have been observed between patients at sites with and without dietetic services with regard to ICU mortality (20.0% vs 25.8%) and 60‐day mortality (22.1% vs 25.8%) (Table 4).

**Table 4:** ICU admission and outcome data.

	Patients at sites with dietetic services	Patients at sites without dietetic services
	(*n* = 140)	(*n* = 97)
Cardiopulmonary bypass used, *n* (%) of patients	120 (85.7)	86 (88.7)
Patient received renal replacement therapy, *n* (%)	45 (32.1)	47 (48.5)
Duration of vasopressor use, h		
Mean ± SD	350. ± 422.9	407.2 ± 482.5
Median (IQR)	228.9 (70.4–523.2)	261.8 (61.4–531.6)
Length of mechanical ventilation, days		
Mean ± SD	17.8 ± 11.9	16.4 ± 11.3
Median (IQR)	14.8 (9.4–19.8)	13.5 (10.4–19.7)
Length of ICU stay, days		
Mean ± SD	20.6 ± 16.1	21.0 ± 17.6
Median (IQR)	14.0 (8.0–28.8)	13.8 (8.4–24.2)
Length of hospital stay, days		
Mean ± SD	28.8 ± 17.1	28.4 ± 17.8
Median (IQR)	23.9 (15.4–40.4)	22.0 (14.1–41.1)
Patient re‐admitted to ICU, *n* (%)	6 (4.3)	3 (3.1)
Patient died within 60 days, *n* (%)	31 (22.1)	25 (25.8)
Patient died during the ICU stay, *n* (%)	28 (20.0)	25 (25.8)

Abbreviations: ICU, intensive care unit; IQR, interquartile range; SD, standard deviation.

## DISCUSSION

The present study revealed that dietetic services in cardiac surgical ICUs are not standard around the globe and need further recognition in critical care treatment of these patients.^13^ ICUs with dietetic services demonstrated a more complete nutrition assessment by history of reduced nutrition intake before ICU admission, more frequently implemented cautious enteral nutrition increment, and parts of a more effective nutrition practice (eg, more patients reaching >80.0% of energy and protein targets on day 7). However, there are still areas for improvement at sites with and without dietetic services.

In our study, dietetic services were available in nearly half of the cardiac surgery ICUs, and staffed full‐time equivalents were low. Interestingly, sites with dietetic services were smaller compared with sites without dietetic services, indicating that larger ICUs do not hold appropriate staffing of dietetic services in relation to their size. Similar information has been received from the nutritionDay initiative: of 880 ICUs surveyed, 593 (67.8%) had dedicated nutritionists on their staff.^9^ Furthermore, in 578 of these ICUs (66.4%), nutritionists were part of a specialized nutrition team.^9^ Despite evidence that dietitians and nutritionists are involved in critical care settings, there is still significant global variation in their integration, both between and within countries. This variation includes dietitian/nutritionist staffing in ICUs, the allocation of full‐time positions, the time spent in the ICU, assignment to different units, and specific roles and responsibilities.^9^, ^14^

With regard to nutrition protocols, it must be noted that a higher percentage of sites without dietetic services had such elements implemented in routine clinical practice compared with sites with dietetic services. This might indicate a structured approach to conducting nutrition practices, especially at those sites not having dedicated professionals taking care of the medical nutrition therapy. However, based on the results received it remains unclear who was responsible for keeping the records and how data were used to guide and adapt nutrition therapy in routine clinical practice. In this context, information about preoperative nutrition status and nutrient intake, which in the present study have been observed being more precise in patients^15^ at sites with dietetic services, are essential when planning and conducting medical nutrition therapy in the critically ill population in general and especially in specific subgroups with a high risk of pre‐existing and iatrogenic malnutrition.^16^

Our study demonstrated that—compared with current recommendations from international guidelines for nutrition therapy in critically ill patients in general—the initiation of both enteral and parenteral nutrition was delayed at sites with and without dietetic services. This observation may be related to patient acuity or hemodynamic instability that precludes feeding. However, specific instances in which enteral feeding may be unsafe or very limited (eg, delayed sternal closure [open chest], use of mechanical circulatory systems,^15^, ^17^ multiple returns to the operating room in the first few days postoperation), which may be very common in post–cardiac surgery patients, have not been captured in the initial International Nutrition Survey. Regardless, postoperative critically ill cardiac surgery patients can be quite unstable, and the presence of dietetic services is important to provide ongoing assessment to determine when it would be safe to start enteral nutrition or, if it is delayed, advocate to start parenteral nutrition. The delay in medical nutrition therapy initiation may also result from the time taken for the dietetic services referral and decision‐making process with the treating intensivists.

Further, although enteral nutrition has predominantly been used in the present study, both parenteral nutrition only and parenteral nutrition in addition to enteral nutrition (enteral nutrition + parenteral nutrition/supplemental parenteral nutrition) were rarely used. However, of those patients receiving a combined enteral nutrition plus parenteral nutrition/supplemental parenteral nutrition regimen, more have been admitted to sites without dietetic services. Although a combined enteral nutrition plus parenteral nutrition/supplemental parenteral nutrition regimen may ultimately improve the delivery of prescribed medical nutrition therapy,^15^, ^17^, ^18^ its use remains debated and not recommended to be initiated early by current international guidelines (more information about the decision to initiate and potential barriers are required in these patients).^19^, ^20^ Therefore, the present data indicate that a dietetic services lead approach may contribute to better adherence to existing guidelines for nutrition therapy in critically ill patients in general.

Our study showed that facilities without dietetic services tend to initiate enteral nutrition close to the current critical care nutrition guidelines, although there was a delay observed in patients at sites with dietetic services.^20^ However, after enteral nutrition initiation, sites with dietetic services implemented more individualized medical nutrition therapy approaches, indicated by the fact that enteral nutrition was initiated at a low rate and gradually increased to the targeted hourly goal rate. Conversely, facilities without dietetic services tend to initiate enteral nutrition at a higher rate, predisposing a higher risk of complications in patients at risk of or with refeeding syndrome.^20^

In our study, facilities with dietetic services were more likely to use nasojejunal feeding tubes. This suggests that dietitians may be more cognizant of the benefits of enteral feeding to maintain the natural physiological use of the gut; hence, they advocated for nasojejunal feeding to enhance enteral tolerance and adequacy, ultimately avoiding the need for prokinetics and the higher costs associated with parenteral nutrition.

Reaching protein targets has been shown to be difficult.^21^ Although the present study revealed some significant efforts at sites with dietetic services to provide more protein, the defined targets were not covered by actual delivery throughout the study period. The fact that defined targets have not been covered by actual delivery has also been observed for energy.

In the context of energy and protein targets, it must be noted that no further details about the underlying methodology or sources of defining nutrition targets in general are available from the initial International Nutrition Survey. It seems obvious that the energy and protein targets defined in the present dataset have been set higher than recommended in most recent international critical care nutrition guidelines without considering a progressive increase throughout the ICU length of stay.^19^, ^20^, ^22^, ^23^ Further, the reference mark of 80.0% chosen to judge on the percentages of energy and protein targets covered by actual intake (Figure 1) was also still defined based on older literature.^24^ However, as the percentages of targets met by actual provision (Figure 1) indicate that the sites were more reluctant with nutrition support and, in general, more closely followed a progressive increase in both energy and protein provision, it could be interpreted that the sites already acted more in line with the newer approaches recommended in current guidelines than with the former standards.

Further, the observation that on day 7 more patients at sites with dietetic services achieved >80.0% of energy and protein targets by actual provision than patients at sites without dietetic services emphasizes efforts to provide nutrition support close to total targets, especially in the later phase of critical illness, and, thus, to adhere with current guidelines.^20^, ^22^, ^23^

With respect to specific enteral nutrition formulations and types of lipids used, the present study reveals differences in patients at sites with vs without dietetic services, which may reflect the heterogeneity in recommendations of international critical care nutrition guidelines^20^, ^22^, ^23^, ^24^ regarding these aspects of medical nutrition therapy. Thus, the results emphasize the need for better evidence‐based clarification on the use of specific (especially immune‐modulating) compositions of enteral and parenteral nutrition products.

In the present study, the use of individualized nutrition and glycemic control protocols was not uniformly implemented at sites with and without dietetic services, representing a further opportunity for improvement of nutrition management in routine clinical practice. As an example, Modir et al.^25^ demonstrated that a cardiac surgery institution‐specific nutrition support protocol created in collaboration with a multidisciplinary team comprised of a registered dietitian, cardiac anesthesia critical care intensivist, cardiothoracic surgeon, and quality director resulted in improved nutrient delivery and reduction in severe nonocclusive bowel ischemia (*P* < 0.001), a frequent complication in cardiothoracic surgical populations.

To enhance the likelihood of implementing optimal nutrition practices in the ICU, it is essential to integrate dietetic services within the multidisciplinary team operating in the ICU, with appropriate staffing and time allocations. Given that tailoring medical nutrition therapy to each patient demands both expertise and time, incorporating appropriately staffed specialized dietetic services into the multidisciplinary team would be advantageous.^3^, ^7^, ^26^ Even if sites with dietetic services may have the advantage of in‐house dedicated nutrition experts who can work with the team to help improve nutrition practice and strategies to optimize nutrition provision—whereas units without dietetic services may not have the expertise, time or manpower—our findings emphasize that increased dietetic services staffing is needed to help improve the suboptimal nutrition practices in critically ill postcardiac surgery patients. In this context, dietetic services could ensure preoperative nutrition screening,^27^, ^28^ implement enhanced recovery after surgery pathways in their assessments,^29^ coordinate postoperative nutrition support–related workflows,^30^ ensure timely malnutrition diagnosis/treatment,^31^ increase the accuracy of malnutrition coding diagnosis for improved reimbursement rate, and, overall, support cardiac surgery nutrition‐related quality improvement efforts to optimize outcomes.^32^

However, with regard to medical nutrition therapy in general it must be noted that aspects such as optimal initiation timing, nutrition route, energy and protein targets, and the use of specific formulas/nutrition supplements in patients after cardiac surgery are still unclear because of limited evidence from clinical trials in this subcohort of critically ill patients. No specific guidelines are available for medical nutrition therapy in patients after cardiac surgery. Thus, further research to define optimal nutrition strategies to guide clinical practices and, thereby, to improve clinical and functional outcome measures in this specific group of critically ill patients is warranted.

### Strengths and limitations

To the best of our knowledge, the present observational study is the first to describe medical nutrition therapy practices in critically ill cardiac surgery patients admitted to ICUs with vs without dietetic services. The prospective, multicenter, observational study design supports the external validity of the findings. However, the results of this exploratory analysis should be interpreted cautiously within the limitations of an exploratory analysis (no a priori statistical analysis plan). Given the exploratory nature of this study, received results should be considered as hypothesis generating only. In this context, it also needs to be considered that the data presented are unadjusted and, therefore, prone to confounding. Further, the variety in institutional structures (eg, personnel involved/responsible for medical nutrition therapy, organization and management of medical nutrition therapy, and guidelines and standard operating procedures used in routine clinical practice) may have led to the variation observed in this study. Further details on patient individual reasons and internal processes that may have affected the initiation timing, choice of route, assessment and definition of energy and protein targets, and actual dosage of medical nutrition therapy (ie, to avoid both underfeeding and overfeeding) are not available from the initial International Nutrition Survey. Thus, no comparison between sites with and without dietetic services can be performed with regard to implemented internal structures and processes. In addition, considering the most recent findings from clinical trials and current recommendations made in international guidelines, the methodological setup of defining 80.0% of energy and protein targets covered by the actual delivery as goal should only be seen as valid in the later study days.

## CONCLUSION

The present findings about daily nutrition practices assessed on cardiac surgery ICUs worldwide demonstrate that significant improvements are required at sites with and without dietetic services. However, the present results also indicate that the involvement of dietetic services allows more tailored approaches for nutrition practice in patients after cardiac surgery. Thus, further efforts to strengthen the role and staffing of dietitians as essential members of an interdisciplinary medical team in the treatment of high‐risk patients, such as those after cardiac surgery, are required to transfer knowledge on appropriate medical nutrition therapy strategies into practice.

## AUTHOR CONTRIBUTIONS

Ellen Dresen and Christian Stoppe equally contributed to the conception and design of the research. Daren K. Heyland contributed to the analysis of the data. Ellen Dresen and Christian Stoppe contributed to the interpretation of the data. Ellen Dresen drafted the manuscript. Danielle E. Bear, Ashley DePriest, Ranna Modir, Omy Naidoo, Charlene Compher, Andrea Ho, Pui Hing Foong, Maria Eloisa Garcia Velásquez, Zheng‐Yii Lee, Charles Chin Han Lew, Gunnar Elke, Jayshil J. Patel, Liam McKeever, Katharina Berschauer, Catarina Rosa Domingues, Juan Carlos Lopez‐Delgado, Patrick Meybohm, Daren K. Heyland, and Christian Stoppe critically revised the manuscript and agree to be fully accountable for the integrity and accuracy of the work. All authors read and approved the final version of the manuscript.

## CONFLICT OF INTEREST STATEMENT

None declared.

## Supporting information

Supporting information.
