# Supplementary material for: Role of dietitians in optimizing medical nutrition therapy in cardiac surgery patients: A secondary analysis of an international multicenter observational study
Source: JPEN J Parenter Enteral Nutr. 2025 Apr 6;49(4):476–87. doi: 10.1002/jpen.2755 (PMC12053139; doi:10.1002/jpen.2755)
Supplement: Supplementary file 1 — Supporting information. [file JPEN-49-476-s001.pdf]

Dresen et al.,

Role of dietitians in optimizing medical nutrition therapy in cardiac surgery patients: A secondary analysis of an international multicenter observational study

Supplementary Tables

Table S-1 Characteristics of participating centers

	Sites with dietetic services (n=8)	Sites without dietetic services (n=5)
Canada	2 (25.0)	0 (0.0)
Germany	0 (0.0)	3 (60.0)
Iran	1 (12.5)	0 (0.0)
Malaysia	1 (12.5)	0 (0.0)
Russia	0 (0.0)	2 (40.0)
USA	4 (50.0)	0 (0.0)

		Sites with dietetic services (n=8)	Sites without dietetic services (n=5)
**Hospital type**, *n (%)*	Teaching	7 (87.5)	5 (100.0)
	Non-teaching	1 (12.5)	0 (0.0)
**Size of hospital (beds)**			
Mean±SD		676.1±346.3	1,143.8±356.4
Range		(160.0-1275.0)	(519.0-1400.0)
**ICU type**, *n (%)*	Closed	4 (50.0)	3 (60.0)
	Open	4 (50.0)	2 (40.0)
**Multiple ICU** *n (%)*	Yes	7 (87.5)	5 (100.0)
**Presence of a medical director**, *n (%)*	Yes	8 (100.0)	5 (100.0)
**Size of ICU (beds)**			
Mean±SD		24.3±6.5	50.4±27.7

		**Sites with dietetic services (n=8)**	**Sites without dietetic services (n=5)**
Range		(14.0-32.0)	(22.0-94.0)
**Full-time position for dietetic services per 10 beds**			
Mean±SD		1.0±0.5	N/A
Range		(0.5-2.0)	N/A
**Number of patients, per site**			
Mean±SD		17.5±5.0	19.4±1.3
Range		(6.0-20.0)	(17.0-20.0)
**Nutrition protocol implemented in clinical practice**, *n (%)*	Yes	4 (50.0)	3 (60.0)

Legend: Values reported as n (%) and mean ± SD (range), respectively.

Abbreviations: ICU, intensive care unit; SD, standard deviation.

Table S-2: Cardiac surgery-specific patient baseline characteristics

	Patients at sites with dietetic services (n=140)	Patients at sites without dietetic services (n=97)
**CCS** *Angina type (n=95 assessed)*		
**No Angina**, n (%)	8 (5.7)	22 (22.7)
**Grade 1**, *n (%)*	16 (11.4)	3 (3.1)
**Grade 2**, *n (%)*	5 (3.6)	8 (8.2)
**Grade 3**, *n (%)*	3 (2.1)	11 (11.3)
**Grade 4**, *(%)*	18 (12.9)	1 (1.0)
**NYHA** *classification (n=123 assessed)*		
**Class 1**, *n (%)*	17 (12.1)	7 (7.2)
**Class 2**, *n (%)*	16 (11.4)	12 (12.4)
**Class 3**, *n (%)*	9 (6.4)	37 (38.1)
**Class 4**, *n (%)*	16 (11.4)	9 (9.3)
**LVEF** *classification (n=213 assessed)*		

	Patients at sites with dietetic services (n=140)	Patients at sites without dietetic services (n=97)
**>50%**, *n (%)*	60 (42.9)	56 (57.7)
**31-50%**, *n (%)*	39 (27.9)	27 (27.8)
**21-30%**, *n (%)*	10 (7.1)	6 (6.2)
**<20%**, *n (%)*	9 (6.4)	6 (6.2)
**Charleson co-morbidity index**		
Mean±SD	1.7±1.8	1.8±2.0
Median (IQR)	1.0 (0.0-3.0)	1.0 (0.0-3.0)
**Functional co-morbidity index**		
Mean±SD	1.7±1.4	1.9±1.6
Median (IQR)	1.0 (1.0-2.5)	2.0 (0.0-3.0)
**Cardiac surgery procedure and data**		
**CABG only**, *n*	32 (22.9)	22 (22.7)

	Patients at sites with dietetic services (n=140)	Patients at sites without dietetic services (n=97)
**Valvular heart surgery only**, *n (%)*	32 (22.9)	27 (27.8)
**Dissecting / ruptured aorta**, *n (%)*	22 (16.3)	18 (18.6)
**Valvular heart surgery + CABG**, *n (%)*	17 (12.1)	10 (10.3)
**Aortic aneurysma**, *n (%)*	1 (0.7)	0 (0.0)
**Others**[§], *n (%)*	26 (18.6)	19 (19.6)
**Baseline cardiac medication**		
**ACE Inhibitors**, *n (%)*	33 (23.6)	42 (43.3)
**Aspirin**, *n (%)*	80 (57.1)	45 (46.4)
**Beta Blockers**, *n (%)*	68 (48.6)	64 (66.0)

	Patients at sites with dietetic services (n=140)	Patients at sites without dietetic services (n=97)
**Statins**, *n (%)*	63 (45.0)	40 (41.2)

Abbreviations: ACE, angiotensin-converting enzyme; ARDS, acute respiratory distress syndrome; CABG, coronary artery bypass grafting; CCS, Canadian Cardiovascular Society; IQR, interquartile range; LVEF, left ventricular ejection fraction; NYHA, New York Heart Association; SD, standard deviation.

§: e.g., further combinations of above-mentioned surgical procedures and additional procedures (e.g. Bentall) etc. that cannot be easily classified

Table S-3: Overall performance (site level)

Overall performance	Sites with dietetic services (n=8)	Sites without dietetic services (n=5)
**Total energy target**, ***kcal/d***		
mean±SD	1,812.7±233.7	1,928.9±148.8
median (IQR)	1,748.4 (1,675.2-1,989.8)	1,976.9 (1,800.3-2,004.9)
**Total energy target**, ***kcal/kg body weight/d***		
mean±SD	23.8±1.4	24.7±2.4
median (IQR)	23.7 (22.5-24.9)	23.4 (23.2-24.8)
**Total protein target, *g/d***		
mean±SD	103.1±20.4	85.3±12.7
median (IQR)	97.1 (89.7-118.2)	80.2 (79.1-90.7)
**Total protein target, *g/kg body weight/d***		
mean±SD	1.4±0.3	1.1±0.2
median (IQR)	1.3 (1.1-1.5)	1.0 (1.0-1.2)
**Total received energy (enteral + parenteral nutrition + propofol), *kcal/d***	878.6±281.6<br>822.1 (725.8-887.7)	813.9±300.4<br>675.1 (626.9-823.3)
mean±SD		
median (IQR)		
**Total received energy (enteral + parenteral nutrition + propofol), *kcal/kg body weight/d***	11.7±3.4	10.7±4.6
mean±SD		

**Overall performance**	**Sites with dietetic services (n=8)**	**Sites without dietetic services (n=5)**
median (IQR)	10.7 (10.0-12.5)	9.0 (8.1-9.6)
**Total received protein (enteral + parenteral nutrition),** ***g/d***		
mean±SD	48.2±18.5	30.1±13.6
median (IQR)	48.6 (33.9-52.2)	26.5 (22.8-27.9)
**Total received protein (enteral + parenteral nutrition),** ***g/kg body weight/d***		
mean±SD	0.6±0.2	0.4±0.2
median (IQR)	0.6 (0.5-0.8)	0.3 (0.3-0.4)
**Received energy from enteral nutrition (enteral nutrition only patients),** ***kcal/d***		
mean±SD	761.6±267.2	624.8±186.5
median (IQR)	778.5 (580.5-868.0)	565.2 (509.3-608.6)
**Received energy from enteral nutrition (enteral nutrition only patients),** ***kcal/kg body weight/d***		
mean±SD	10.0±3.3	8.3±3.0
median (IQR)	9.9 (8.2-11.6)	7.4 (7.2-7.5)

Overall performance	Sites with dietetic services (n=8)	Sites without dietetic services (n=5)
**Received protein from enteral nutrition (enteral nutrition only patients),** ***g/d*** *mean±SD* *median (IQR)*	45.5±18.8 39.4(31.3-55.5)	25.5±7.3 22.9 (22.3-25.9)
**Received protein from enteral nutrition (enteral nutrition only patients),** ***g/kg body weight/d*** *mean±SD* *median (IQR)*	0.6±0.2 0.5 (0.4-0.7)	0.3±0.1 0.3 (0.3-0.3)
**Total adequacy of energy (enteral + parenteral nutrition),** ***%*** *mean±SD* *median (IQR)*	49.9±13.6 45.4 (43.8-53.8)	43.8±18.5 35.3 (35.2-41.9)
**Total adequacy of protein (enteral + parenteral nutrition),** ***%*** *mean±SD* *median (IQR)*	47.5±14.2 44.3 (39.0-57.5)	37.2±22.6 26.8 (25.7-35.4)
**Adequacy of energy from enteral nutrition (enteral nutrition only patients),** ***%*** *mean±SD* *median (IQR)*	43.0±13.9 41.1 (34.6-53.2)	33.7±12.0 31.8 (26.0-32.7)

**Overall performance**	**Sites with dietetic services (n=8)**	**Sites without dietetic services (n=5)**
**Adequacy of protein from enteral nutrition (enteral nutrition only patients), *%***		
mean±SD	45.2±16.3	31.1±13.0
median (IQR)	41.5 (33.1-60.6)	26.9 (25.2-28.5)

Abbreviations: IQR, interquartile range; SD, standard deviation.

Table S-4: Reasons enteral nutrition feeds interrupted

Reasons interruption	Sites with the presence of dietetic services; n (%) of patient days[*] (n=1396)	Sites without the presence of dietetic services; n (%) of patient days[*] (n=992)
Fasting for endotracheal extubation/intubation/trach procedure	75 (5.4)	45 (4.5)
Fasting for operating room procedures	19 (1.4)	30 (3.0)
Fasting for other bedside procedure	27 (1.9)	28 (2.8)
Enteral nutrition intolerance[**]	62 (4.4)	63 (6.4)
No enteral access available / enteral access lost, displaced or malfunctioning	20 (1.4)	6 (0.6)
Trial of oral intake	5 (0.4)	15 (1.5)
Inotropes, vasopressor requirement	3 (0.2)	28 (2.8)

**Reasons interruption**	**Sites with the presence of dietetic services; n (%) of patient days[*] (n=1396)**	**Sites without the presence of dietetic services; n (%) of patient days[*] (n=992)**
Subject deemed too sick to continue enteral feeding	6 (0.4)	5 (0.5)
Fasting for radiology suite procedure	20 (1.4)	14 (1.4)
Fasting for administration of medications	2 (0.1)	0 (0.0)
New contra-indication to enteral nutrition	1 (0.1)	3 (0.3)
Other***	20 (1.4)	9 (0.9)

* Related to n=2388 of total patient days

** Increased gastric residuals (6), vomiting/emesis (8), increased abdominal girth or abdominal distension (7), subjective discomfort, diarrhea or other (10)

** including gastrointestinal bleeding (2), Patient agitation (2), Unknown (2), Acute arterial bleeding (1), CPAP/vent weaning (1), Hydrotherapy (shower table) (1), Patient on CPAP (1)

Abbreviations: CPAP: continuous positive airway pressure.

Table S-5: Composition of enteral formulas

Enteral formulas	Patients at sites with dietetic services (n=127)	Patients at sites without dietetic services (n=93)
Arginine enriched formula	1 (0.8%)	0 (0.0%)
Fish oil enriched formula	19 (15.0%)	0 (0.0%)
Glutamine enriched formula	0 (0.0%)	0 (0.0%)
Polymeric formulas	105 (82.7%)	93 (100.0%)

Table S-6: Use and type of lipids

Lipids received	Patient days at sites with dietetic services (n=84)	Patient days at sites without dietetic services (n=86)
Soybean oil based	11 (13.1%)	2 (2.3%)
Medium-/Long-chain triglycerides physical mixture	0 (0.0%)	73 (84.9%)
Mixture of medium-chain triglycerides, soybean, olive, and fish oil	39 (46.4%)	6 (7.0%)
Fish oil based	0 (0.0%)	1 (1.2%)
Lipid free	34 (40.5%)	0 (0.0%)

Table S-7 Glycemic control

Variable	Patients at sites with dietetic services (Patient level data) n=140	Patients at sites without dietetic services (Patient level data) n=97	Sites with dietetic services (site level data) n=8	Sites without dietetic services (site level data) n=5
Presence of glycemic control protocols, n (%) of sites	N/A	N/A	6 (75.0)	3 (60.0)
Blood glucose levels in the moring (mmol/L)				
mean±SD	8.2±1.8	7.8±1.2	7.9±0.4	7.5±0.4
median (IQR)	7.9 (7.2-9.0)	7.6 (6.9-8.2)	8.0 (7.7-8.2)	7.6 (7.3-7.8)
Periods of hyperglycemia (%)	16.0	12.0	10.7	2.7
Periods of hypoglycemia, (%)	1.3	0.7	0.0	0.0

Abbreviations: IQR, interquartile range; SD, standard deviation.
